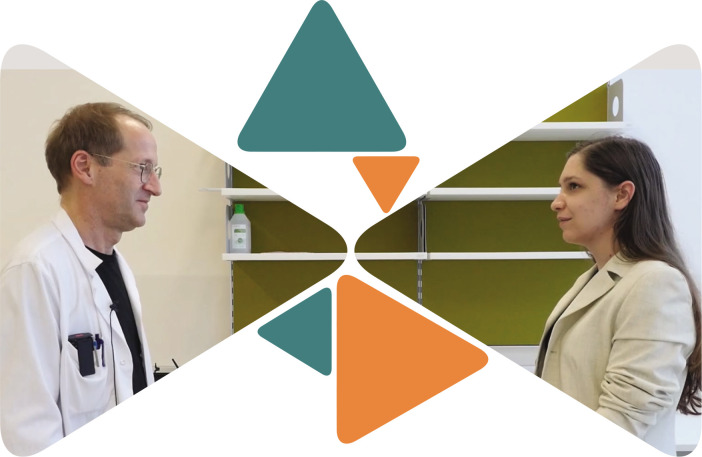# Peter Lackner – Adapted interviews from Neurotrauma Treatment Simulation Center (NTSC) – Vienna, 2022

**DOI:** 10.25122/jml-2022-1020

**Published:** 2022-07

**Authors:** Andreea Strilciuc, Stefana-Andrada Dobran, Alexandra-Mihaela Gherman

**Affiliations:** 1. RoNeuro Institute for Neurological Research and Diagnostic, Cluj-Napoca, Romania; 2. Sociology Department, Babes-Bolyai University, Cluj-Napoca, Romania

**Interviewee: Peter LACKNER**

**Interviewer: Stefana DOBRAN**

**S.D**.: Hello, Professor Lackner! Thank you for having us here today, at the Neurotrauma Treatment Simulation Center. We are on the last day and it has been quite an experience. I have some questions for you, starting with… can you tell us a bit about yourself and your background and focus area?

**P.L**.: You're well welcome. I'm very happy that we were able to start with this first Neurotrauma Treatment Simulation Center (NTSC) in Vienna, which so far is very interesting for me and there has been a big and a good community from all over the world coming to visit Vienna and to get an exchange with us and improve neurotrauma treatment all over the world. So, concerning me, I'm actually a neurologist, I'm a neurointensivist, and I have a big background in neurocritical care. Before I moved to Vienna, I was working at the Medical University in Innsbruck, in the Neuro Critical Care Department where we were responsible for neurotrauma treatment and now that I've moved to Vienna, I'm the head of the Department of Neurology here, in Floridsdorf and also in Klinik Penzing. Klinik Floridsdorf is specialised in acute neurology which is focusing on cerebrovascular diseases, but also on mild and moderate TBI which we frequently take care of in this hospital. And Klinik Penzing is an early rehabilitation ward where patients with severe neurological deficits, like severe stroke, intracerebral bleeding, and also traumatic brain injury are being taken care of after the acute setting in the early rehabilitation setting. So, I'm in the lucky position of having two departments where we are able to cover the whole chain of trauma care. On the one hand, the acute setting and, on the other hand, the early rehabilitation setting.

**Figure:**
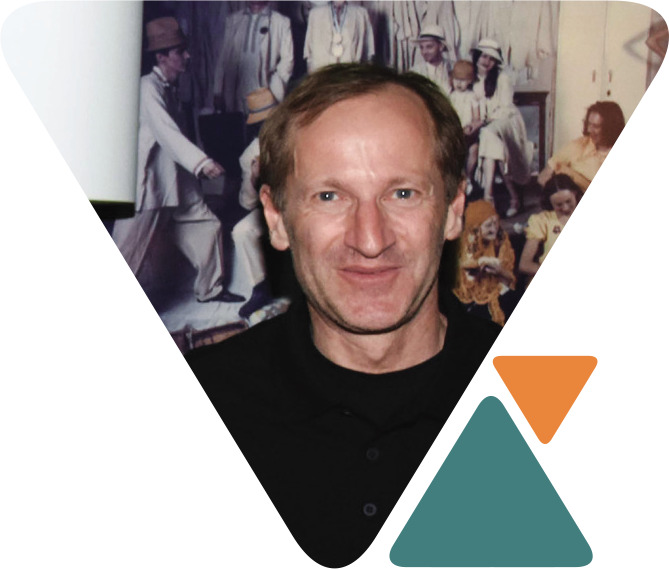


**S.D**.: Right. Thank you! And what motivated you to be part of the coordination team of this program?

**P.L**.: Actually, the idea of performing a simulation center came up, I think, two years ago, when we were talking about stroke simulation [...] I was talking with colleagues at an AMN (Academy for Multidisciplinary Neurotraumatology) meeting, about our stroke simulation program that we had instituted in the Klinik Floridsdorf, because we said we have to have stroke simulation to improve our time from door to needle treatment, our door to needle time, so we set a stroke simulation program here, in the Klinik Floridsdorf and then the idea came up that we can have it also for TBI. And in TBI there are many treatment critical points. For instance, door to CT time, or door to reversal anticoagulation treatment, and we thought that this would be a good chance to have a simulation program set up. I discussed it with my colleagues here, in Vienna, and many of them said 'We have similar things in mind, we have many different other processes which we could improve if you simulate it.' And then, all of a sudden, colleagues from traumatology, colleagues from neurosurgery, from anesthesiology, from rehab centers, got on board and we were able to set up [...] for the first time, this Neurotrauma Simulation Center which we are very proud of and, hopefully, also the participants will like it.

**S.D**.: Right. So, it was truly a multidisciplinary approach.

**P.L**.: Yes.

**S.D**.: Alright. Can you tell us a bit about how your experience shaped your opinion on the management of neurotrauma?

**P.L**.: Actually, as I've already said, there are so many processes in the complex treatment of neurotrauma. First of all, neurotrauma in itself is so complex, the pathophysiology of neurotrauma is so complex that you need a team, you need a big team to do the best for your patient. And this came into my mind already when I started with neurocritical care - that's over 20 years ago when I first was there as a young doctor and I saw the complexity of the care chain for neurotrauma patients. I realised that you have to be a team player. You have to have leaders, but you also have to have team players and the neurotrauma chain of care is only as strong as the weakest part of it is. So, we have to improve the weakest part of the chain, of the whole chain, and that is one of the reasons why I am so enthusiastic to improve the multidisciplinary approach.

**Figure:**
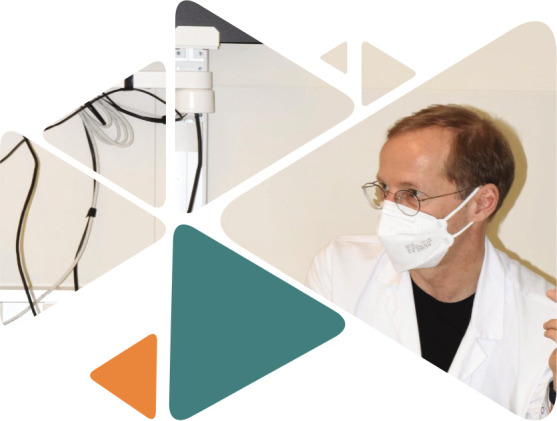


**S.D**.: Alright. Thank you! Are you familiar with any similar programs running at the moment?

**P.L**.: It is not that I know of similar programs. So, there are similar programs in stroke, but as far as I know, there is no other program comparable to this program in TBI.

**S.D**.: It's good, we are innovating the field.

**P.L**.: Hopefully, yes.

**S.D**.: If you have to identify 3 challenges in the approach to TBI, which would come to mind?

**P.L**.: First of all, we need data. If we do not have data, we do not know what our most relevant problems are and of course, if we perform any action to change any mode of action we are doing, so we don't know if we are successful. So, first of all, we need data, and that's why we also, in the AMN, are so enthusiastic and so hardworking for providing a new registry which is easy [...] manageable, which only takes short time and includes important quality indicators for the processes you have in the TBI treatment. So, I think we need data and then, we need to improve our collaborations. Of course, in many countries over the world, we are lacking resources, and we lack expensive structures. But in many parts of the world, we can improve what exists there if we just improve our multidisciplinary collaboration. [...] First thing, data, the second thing, improve collaboration and the third thing, step wisely, improve also, of course, the availability of structures of health care in the whole trauma chain. So, not only in acute care of trauma, in pre-clinical care of trauma, but also in the clinical care of trauma and finally, in the rehabilitation setting. So again, if we do not have the structures, we could start improving the collaboration between the different chains of trauma care. So, data, multidisciplinarity, collaboration and, last but not least, improved structures.

**S.D**.: Alright. Regarding the treatment and rehabilitation process for patients suffering from neurotrauma, which organizational issues do you think are the most pressing?

**P.L**.: In the treatment process, what we could improve and have the most benefit out of, actually, are treatment paths. To establish a pathway which should be our standard, so we can stick to this pathway. And we need to have standard operating procedures with which we can handle the patient from one organization, or from one phase of the trauma care to the other. Or, from pre-clinical to clinical, from clinical to outpatient care. So, if we have a defined pathway which we can attire, I think we can [...] reach a big step in the improvement of trauma care.

**S.D**.: Alright. From your point of view, which are the main limitations in the approach to cognitive, behavioural and depressive disorders related to neurotrauma cases?

**P.L**.: That we do not detect them. So, I think the biggest drawback is that we do not have a standard assessment of neurocognitive deficits. So, if we do not diagnose them, how can we treat them?! First of all, we will have to set up defined protocols which we are sticking to and attire, to diagnose cognitive or neuropsychiatric health disorders, and then we can think of how we can change the progress [...] of aiding people or supporting people with the problems and even treating those disorders.

**S.D**.: Alright. Could you please describe to us one of the most intriguing, interesting, or complicated neurotrauma cases in your career? [...] One that you remember above all…

**P.L**.: Any neurotrauma case is very complicated. So, actually, we once had a case when part of the brain prolapsed out of the skull, it was a very young patient, I think he was 16. With the combined effort of neurosurgeons who were the main actors in the acute phase and then, afterwards, the neurocritical care unit and afterwards, the neurorehabilitation phase... So, imagine, prolapsing part of the brain out of the skull and, two years later, having a patient who was able to walk again... My message *is - sometimes it takes longer, but it's really worth the investment*.

**S.D**.: That is really impressive. Online communities in the medical field are becoming more and more visible. What is your take on the importance of these communities for the advancement of medicine and research?

**P.L**.: They are definitely the drivers of structural chains. So, our interdisciplinarity and [...] internationality is very important, in my opinion, because each country can learn from each other. It is [...] what history shows us, the drivers of change in medical support and [...] in the way we think in medicine. So, especially the AMN, being a multidisciplinary community, is a very important driver of change in the field of TBI, in my opinion.

**Figure:**
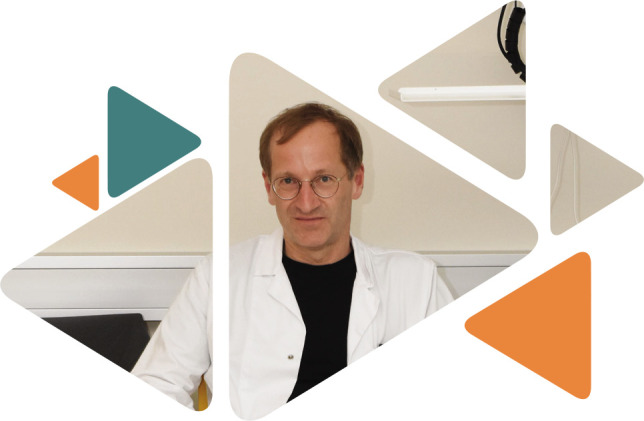


**S.D**.: Alright. And, what do you consider the impact of COVID-19 pandemic was on neurotrauma management and on the caseload as well?

**P.L**.: On the caseload, we first had a decrease because we had these lockdowns all over the world and if people stayed at home, they experienced less trauma, of course. Then, we had an increase back again so, the last two years were really challenging for us because we were always competing for intensive care beds, let's say. If we had a lockdown, we had more Covid patients than after the lockdown. Covid went down and neurotrauma went up again.

So, we had, I would say, two years without any pause. And that's the most dangerous, in my opinion, the most dangerous and challenging situation we are facing now. We are facing a human resource problem. We are really facing a human resource problem, after two years of combined efforts, many of our colleagues and especially nurses, are on the brink of burnout. [...] We really have to take care of our human resources in the hospital.

**S.D**.: Alright. Thank you! And the last question would be, what do you think are the main challenges or obstacles that would affect a proper long-term follow-up with the patients with neurotrauma?

**P.L**.: Proper long-term follow-up… [...] So, the problem we have is, as the treatment phases of trauma at least include 3, better 4 different phases, the communication between these phases is still non-existent in many countries worldwide, including Austria. So, we will have to set up protocols where the data is collected in the different phases and where we can monitor the whole phase of trauma care in one patient.

And then, again, look at the quality indicators and learn from the process, and learn from each individual patient and improve the process, but this would only be possible, in my opinion, if we had the data on it.

**S.D**.: Alright. This was my last question. Thank you very much, Professor Lackner for the interview and thank you for having us here today.

**P.L**.: You're welcome. [...]

Watch the extended interview on the AMN Website: https://brain-amn.org/ntsc-interviews-series-peter-lackner/

**Figure:**